# The Hemiarthroplasty of the Proximal Interphalangeal Joint in Post-traumatic Degenerative Disease: A Case Report

**DOI:** 10.7759/cureus.65326

**Published:** 2024-07-25

**Authors:** Jakub Florek, Filip Georgiew, Oles Petrovych

**Affiliations:** 1 Department of Orthopedics and Traumatology, Rydygier Hospital, Brzesko, POL; 2 Faculty of Health Science, University of Applied Sciences, Tarnów, POL

**Keywords:** hand injuries, arthroplasty, pip, proximal interphalangeal joint, osteoarthritis

## Abstract

Fractures of the proximal interphalangeal (PIP) joint with fragment displacement should be promptly repaired after injury, though this does not ensure the return of pre-injury finger function. This article presents the case of a 29-year-old patient who sustained an injury to the fourth finger of his right hand, resulting in an open fracture of the distal and shaft of the proximal phalanx involving the PIP joint and partial damage to the finger extensor mechanism. Immediately post injury, the fracture was realigned and stabilized with Kirschner wires (K-wires). Three years later, due to post-traumatic degenerative disease, the patient required further surgical intervention and was diagnosed with type III according to the modified Kellgren and Lawrence scale. The decision was made to perform a partial arthroplasty of the PIP joint. The implantation of the PIP prosthesis in a patient with post-traumatic degenerative disease can restore the correct range of flexion movement, realign the fourth digit, and eliminate pain. However, this treatment method may pose a risk of a slight limitation in the range of extension motion in the joint.

## Introduction

Post-traumatic osteoarthritis of the proximal interphalangeal (PIP) joint is characterized by pain, limited mobility, swelling, and deformities of the affected fingers [1]. Approximately 12% of all degenerative joint diseases (osteoarthritis) result from trauma [2]. Nonsurgical treatments may alleviate pain, but their effectiveness diminishes as the disease progresses [3,4]. Surgical interventions can permanently eliminate pain and swelling in the advanced stages of the disease and, in some cases, improve the joint range of motion. Surgical treatment methods for post-traumatic PIP joint disease include arthrodesis, arthrotomy with Reg-Joint (a bioresorbable implant for small joint arthrosis), arthroplasty using a vascularized graft from the toe joints, arthroplasty using a non-vascularized graft from the hamate bone, and arthroplasty using silicone prostheses [5-7].

Fractures in the proximal interphalangeal (PIP) joint with fragment displacement should be repaired as soon as possible post injury; however, this does not guarantee the restoration of the finger's pre-injury function [1]. Current evidence-based medicine reports indicate that no ideal treatment method exists that combines complete pain relief with the preservation of the preoperative range of motion [1]. The medical care provided to the patient discussed in this case may offer valuable insights into the surgical treatment of this type of condition.

## Case presentation

**Figure 1:**
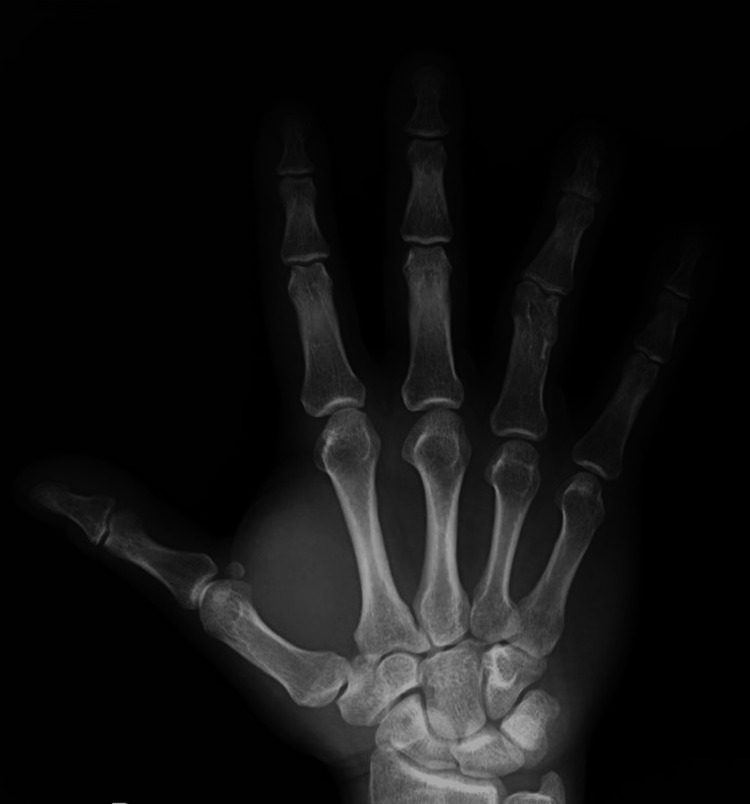
X-ray of the right hand in AP projection, three years after the initial treatment with Kirschner wires AP: anteroposterior

**Figure 2:**
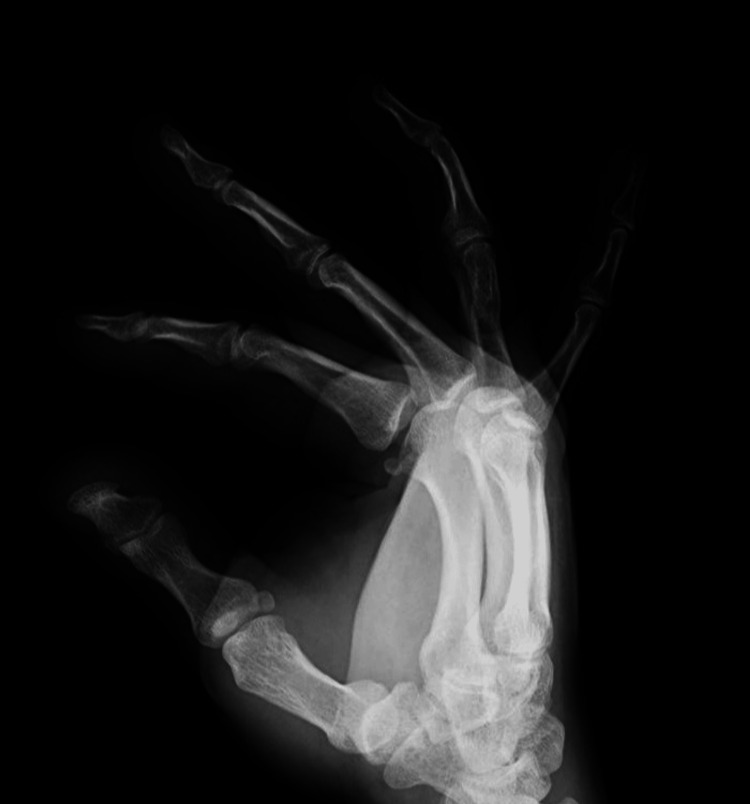
X-ray of the right hand in lateral projection, three years after the initial treatment with Kirschner wires

The article describes the case of a 29-year-old patient who sustained an injury to the fourth finger of his right hand, resulting in an open fracture of the distal and shaft of the proximal phalanx with the involvement of the PIP joint and partial damage to the finger extensor. Immediately post injury, the fracture was reduced and stabilized with Kirschner wires (K-wires), and the finger extensor was sutured. Seven weeks after the procedure, the K-wires were removed, and rehabilitation commenced. By the fourth month, the range of extension in the PIP joint returned to normal, the range of flexion reached 10 degrees, and pain intensity was recorded at 2 on the visual analog scale (VAS). After 10 months, the patient developed a flexion contracture and an ulnar deformation of the PIP joint, and rest pain increased to level 6 on the visual analog scale (VAS). Three years after the initial treatment, he was qualified for further surgical intervention due to the post-traumatic degenerative disease of the PIP joint of the fourth finger. The patient was diagnosed with type III according to the modified Kellgren and Lawrence scale (Figures 1, 2).

**Figure 3:**
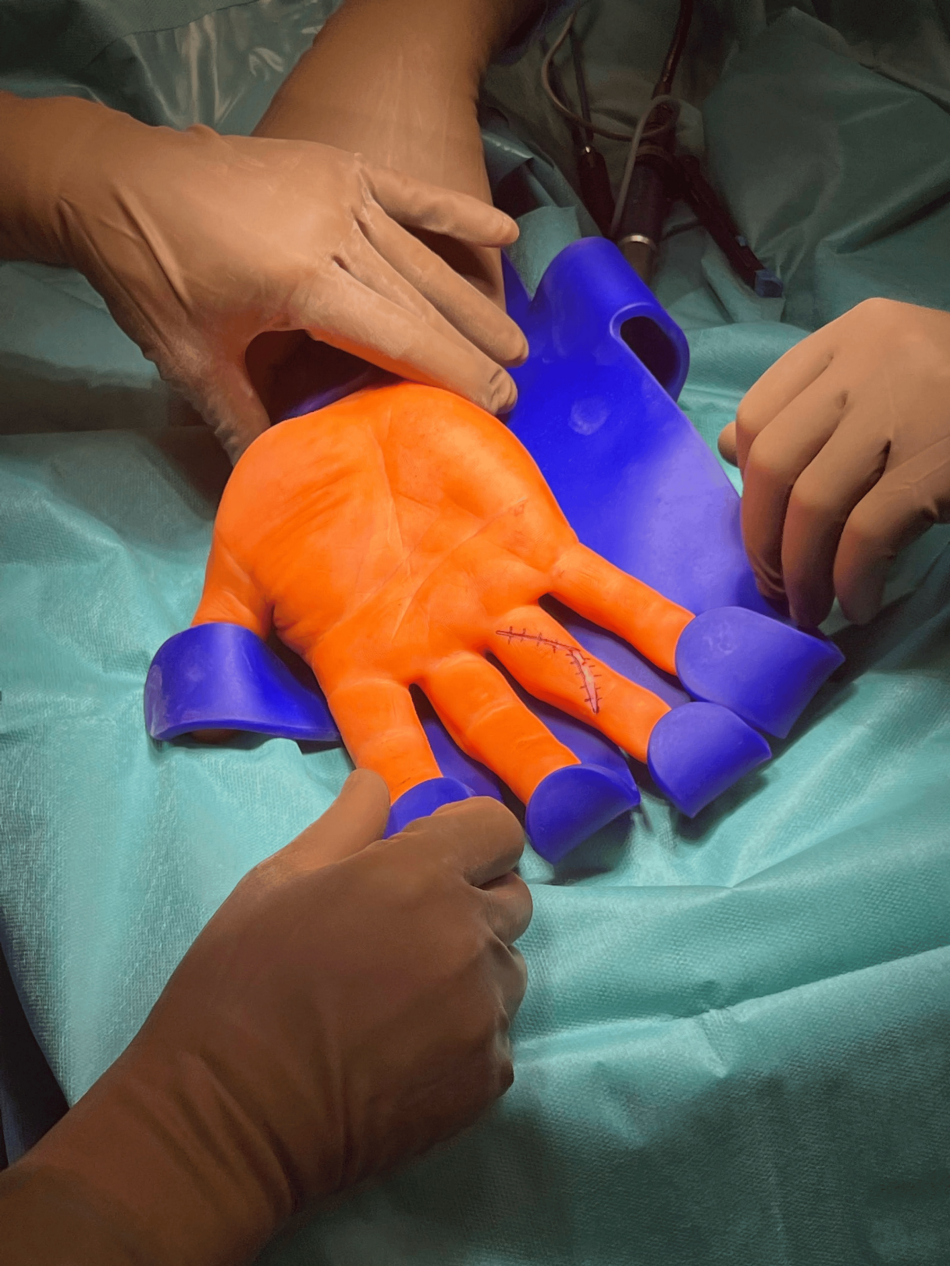
V-shaped skin incision

Clinical examination confirmed the presence of post-traumatic elbow deformation of the PIP joint at 10 degrees, an extension deficit of 15 degrees (flexion contracture), and the limitation of flexion mobility to 60 degrees. The intensity of rest and functional pain was rated at 6 on the VAS. A decision was made to perform a partial arthroplasty of the PIP joint using the CapFlex prosthesis (KLS Martin Group, Tuttlingen, Germany). The CapFlex PIP prosthesis, composed of a combination of metal and polyethylene, provides a high degree of stability and mobility for the proximal interphalangeal joints. A palmar approach was utilized with a "V"-shaped incision running over the proximal and middle phalanges of the fourth finger of the right hand (Figure 3).

**Figure 4:**
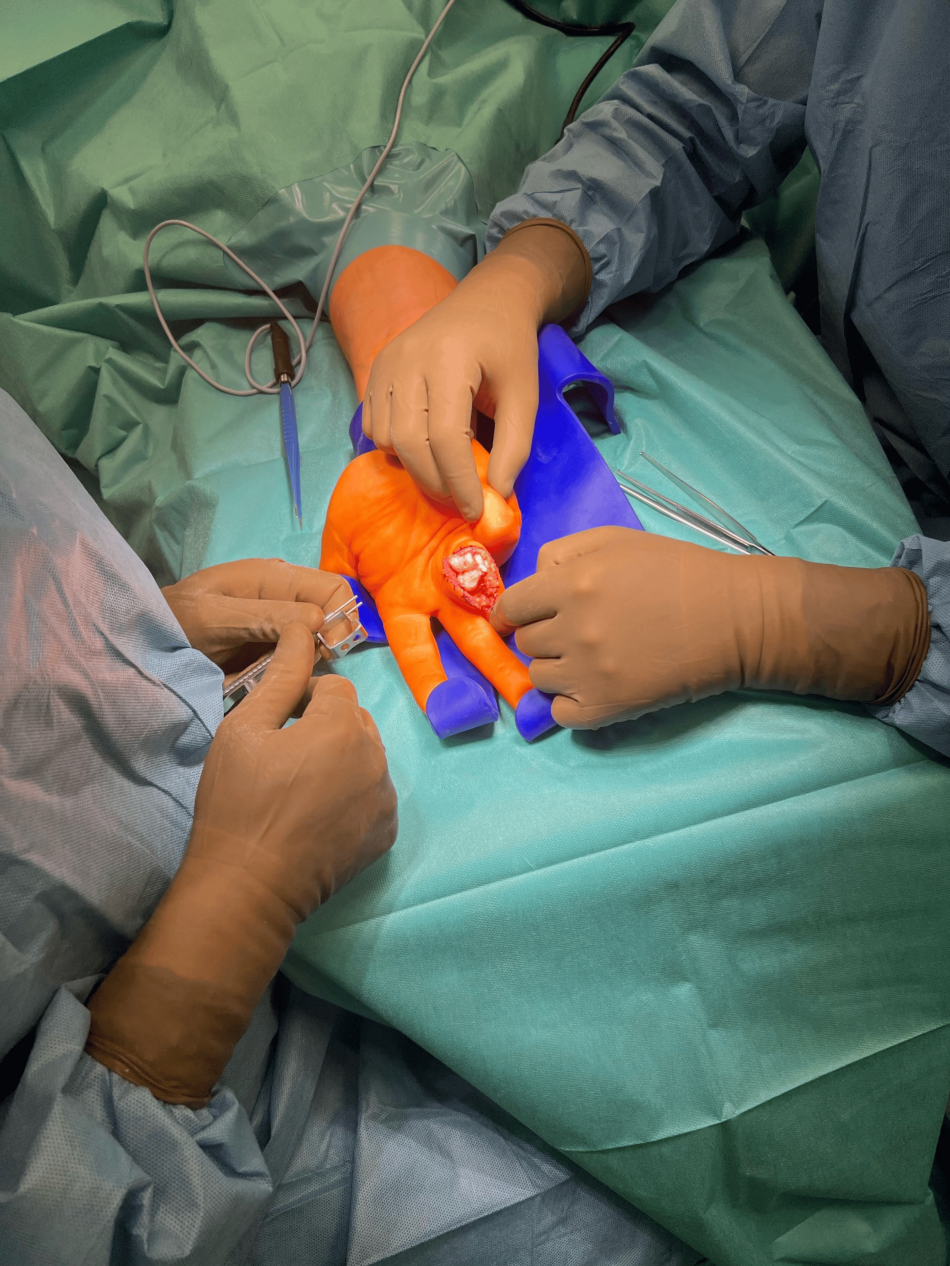
"Shotgun" joint dislocation

In the first stage, the neurovascular bundle and flexor tendons of the fourth finger of the right hand were carefully dissected. The tendons were then moved to the ulnar side of the hand. Subsequently, the palmar plate was exfoliated, and the PIP joint was dislocated forward using the "shotgun" method (Figure 4).

**Figure 5:**
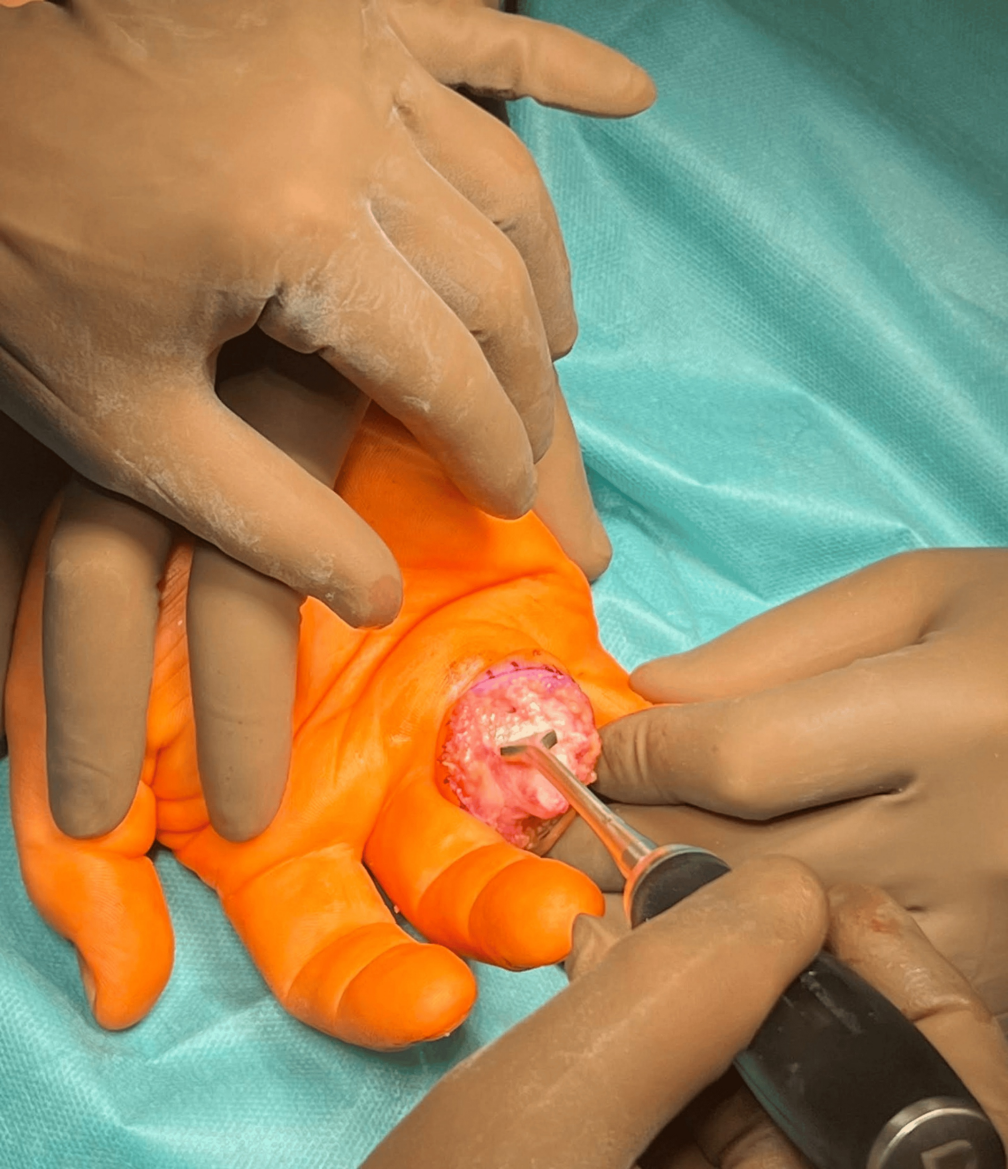
Preparation of the distal end of the post-traumatically altered proximal phalanx

**Figure 6:**
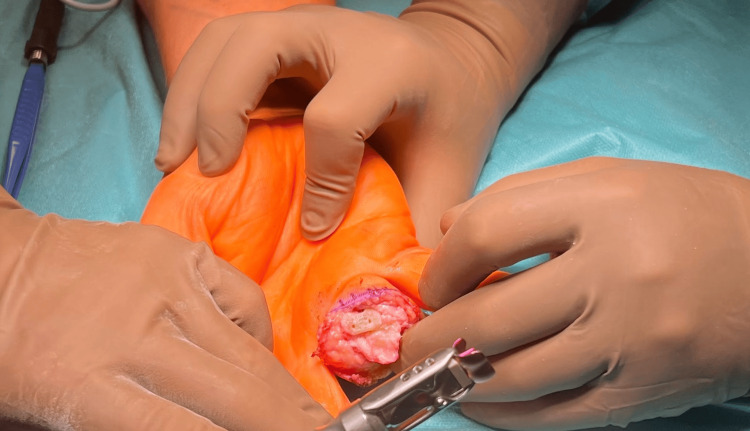
Preparation of the distal end of the post-traumatically altered proximal phalanx

After performing the maneuver, a linear incision was made at the post-traumatic change of the distal end of the proximal phalanx. Precise microcuts were made using a micro saw to prepare the operating bed for the trial fitting of the prosthesis, preserving the entire collateral ligaments of the PIP joint of the fourth finger (Figures 5, 6).

**Figure 7:**
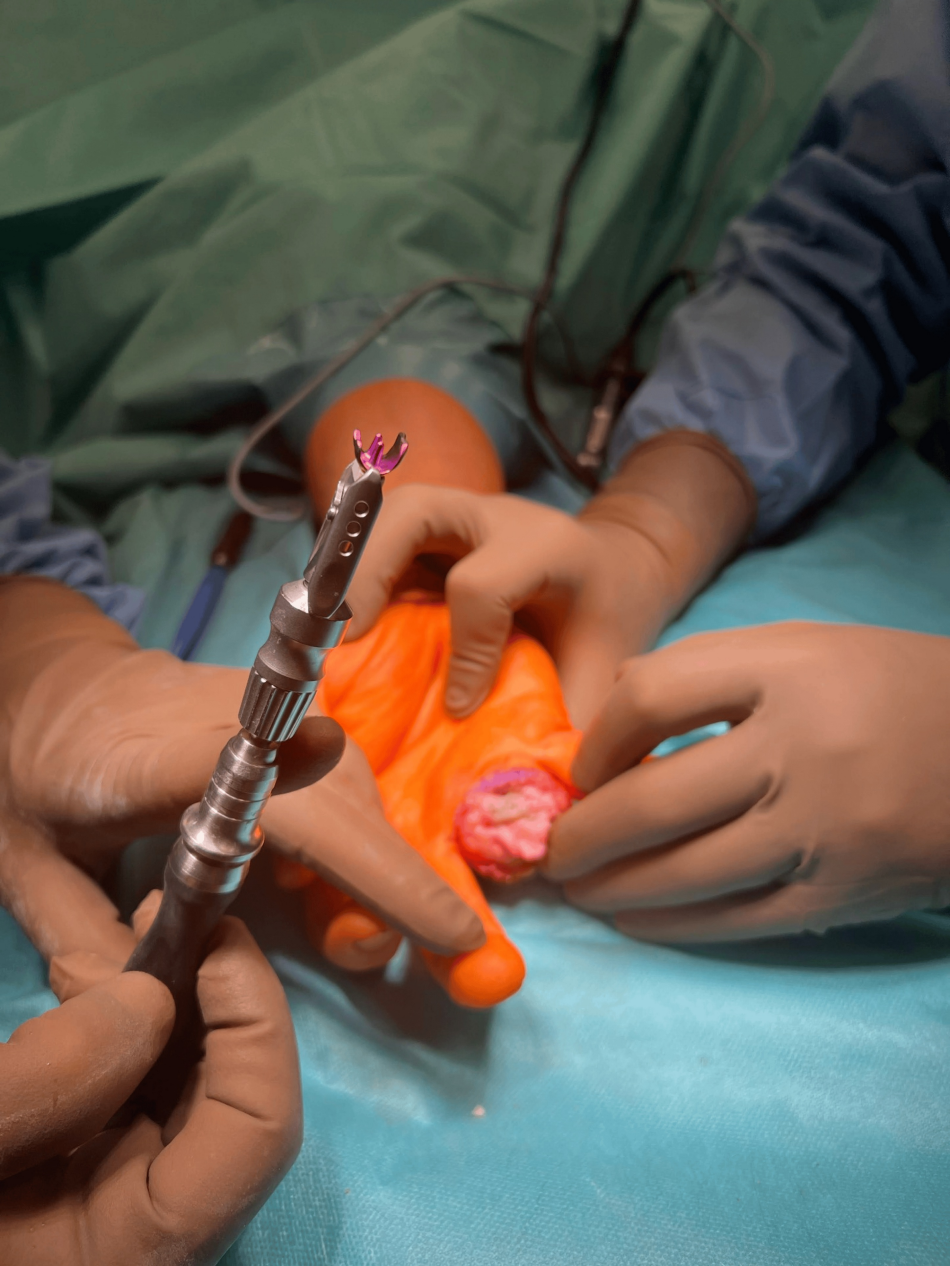
Implantation of the "trim-on" CapFlex PIP prosthesis of the proximal phalanx PIP: proximal interphalangeal

**Figure 8:**
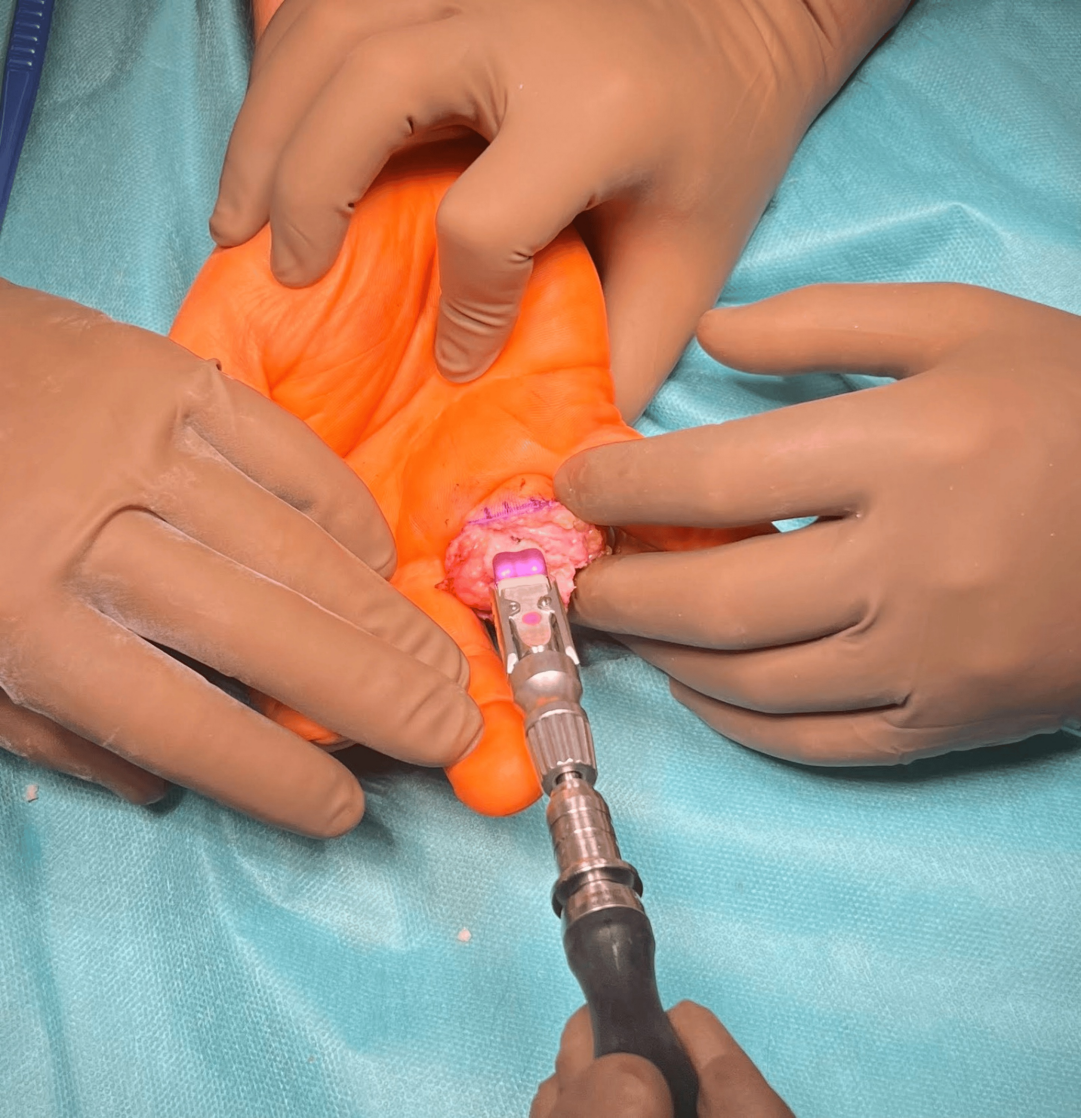
Implantation of the "trim-on" CapFlex PIP prosthesis of the proximal phalanx PIP: proximal interphalangeal

A measuring prosthesis was implanted on the prepared base (Figures 7, 8).

**Figure 9:**
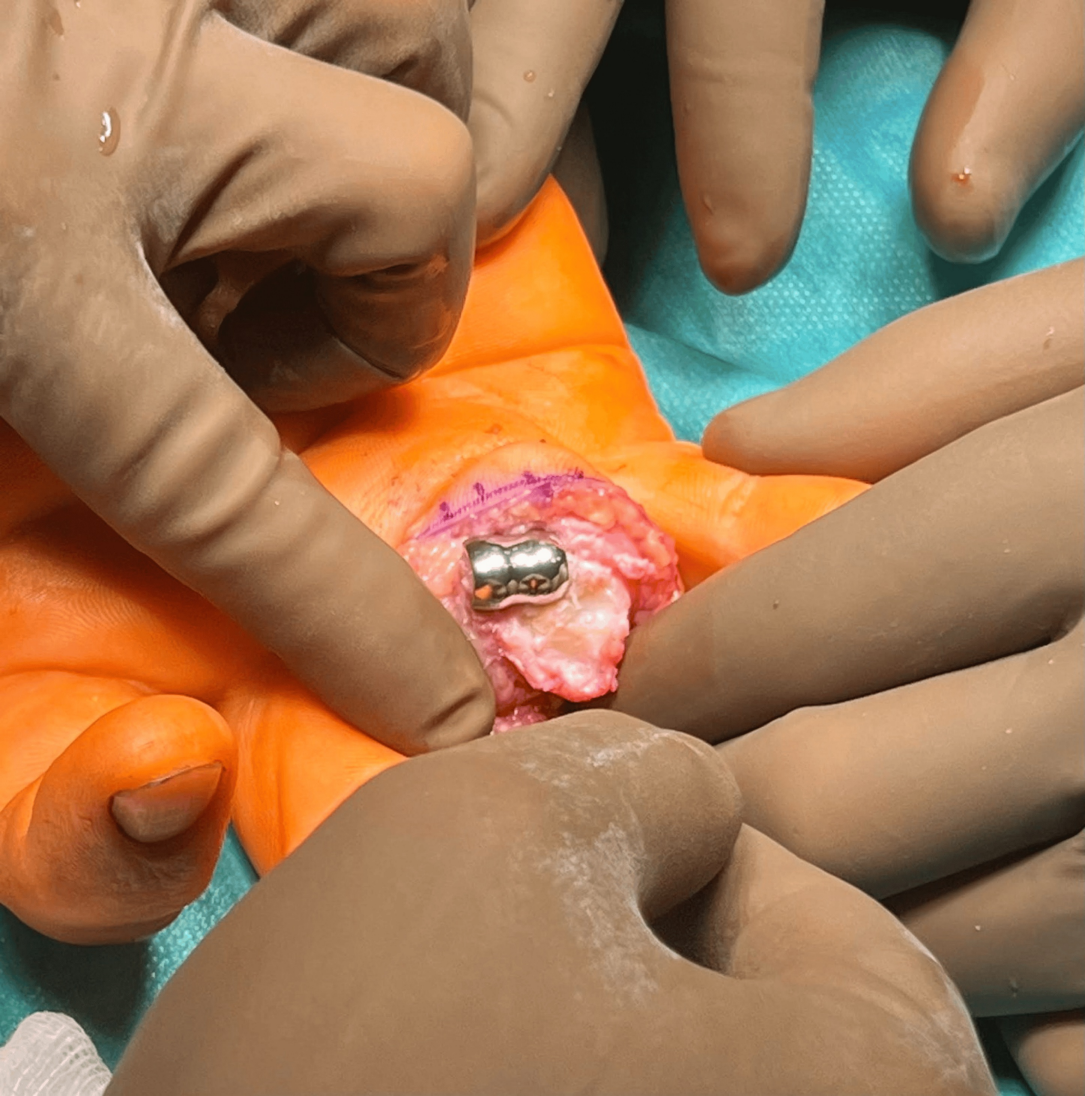
Implantation of the "definitive" CapFlex PIP prosthesis of the proximal phalanx PIP: proximal interphalangeal

In the next stage, the CapFlex prosthesis implant for the PIP joint was placed using the pre-fit method (Figure 9).

**Figure 10:**
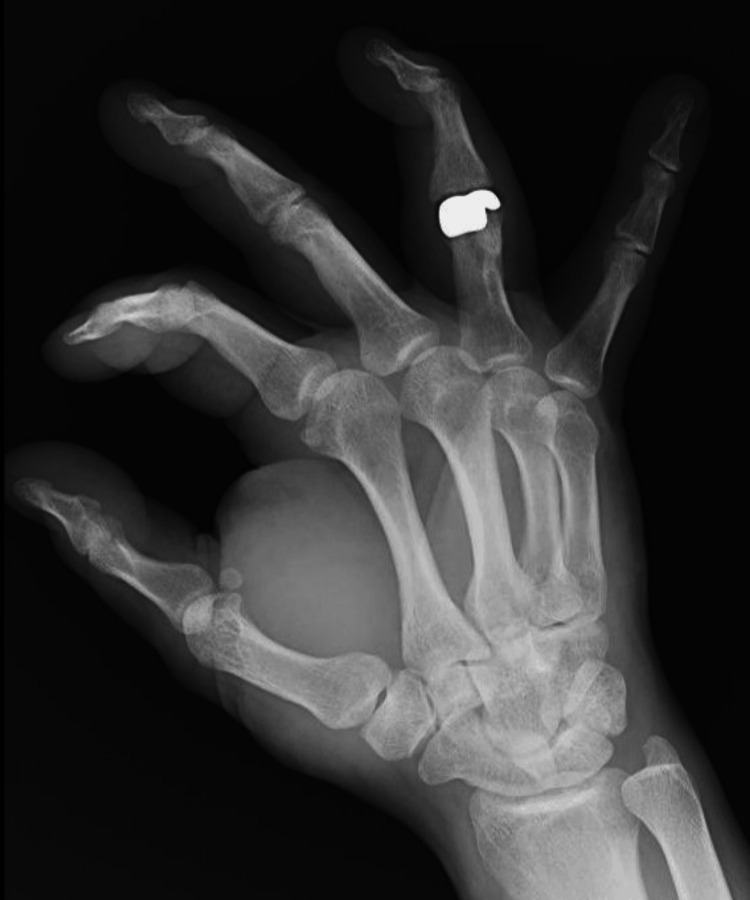
Lateral X-ray obtained eight months after surgery

**Figure 11:**
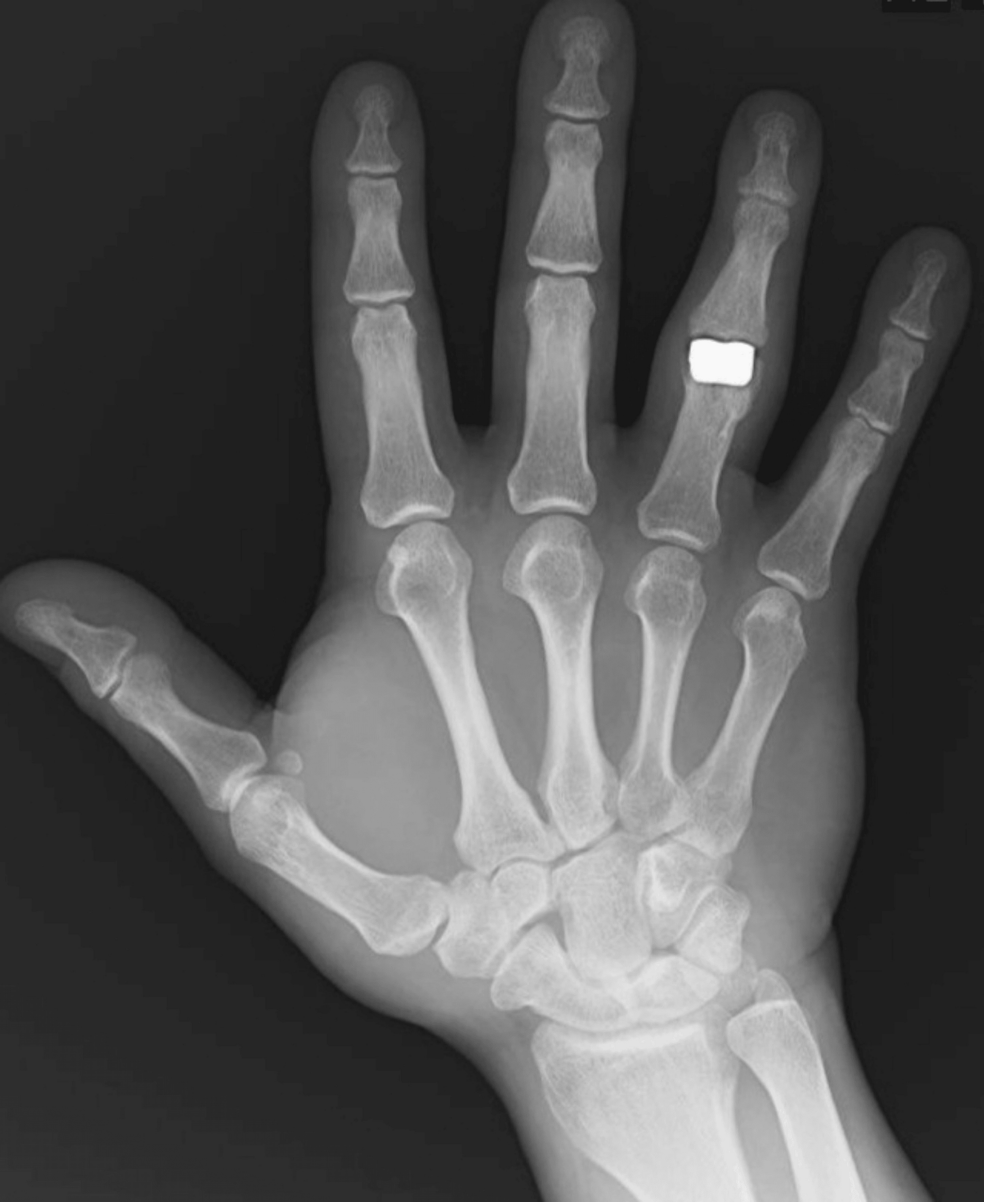
Anteroposterior X-ray obtained eight months after surgery

**Figure 12:**
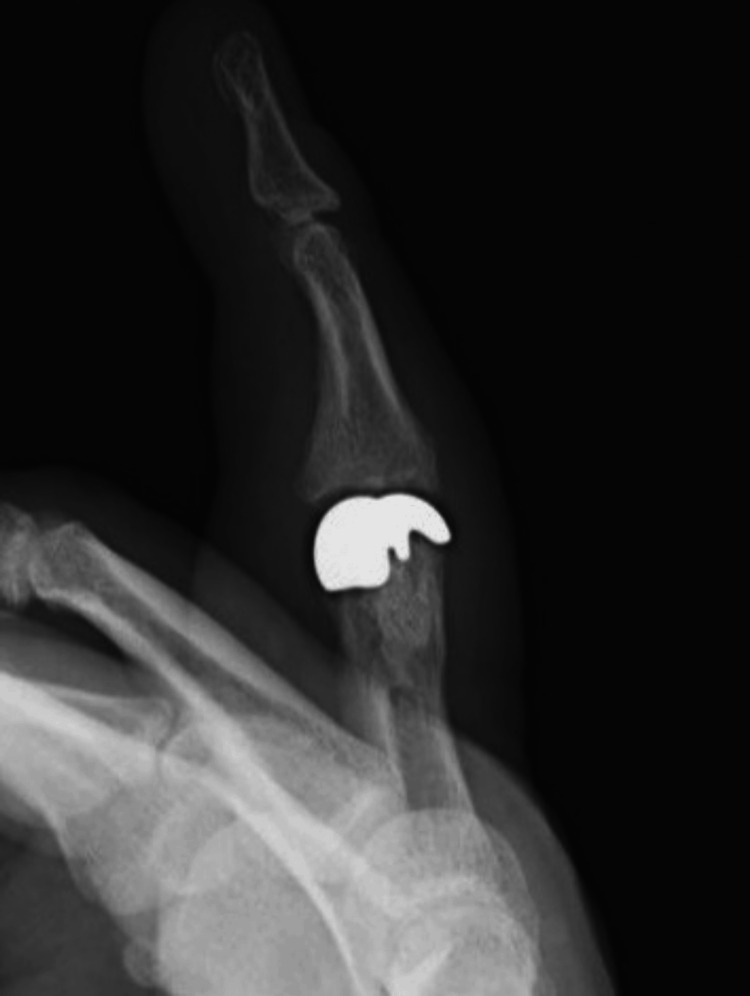
PIP-aimed X-ray obtained eight months after surgery PIP: proximal interphalangeal

At the end of the procedure, the stability of the prosthesis and the range of passive mobility from 0 to 100 degrees were checked. Fingers III and IV were immobilized with a plaster splint on the palmar side. On the third day post operation, the patient had passive mobility of 0-90 degrees. After two weeks, the immobilization was removed, allowing the movement of the fourth finger of the right hand, and the patient's rehabilitation process began. Four weeks after the procedure, improvement in the active flexion range of motion to 50 degrees was confirmed, with an extension deficit remaining at 5 degrees. Eight weeks post procedure, the range of active flexion movement increased to 80 degrees, while a 5-degree extension deficit persisted. The intensity of rest and functional pain was 0 on the VAS. The correction of the fourth axis of the fourth finger of the right hand was achieved. Three months post surgery, the patient returned to work. Figures 10-12 show the follow-up X-ray eight months after surgery.

## Discussion

Conservative treatment may yield favorable outcomes in early-stage degenerative disease; however, its efficacy diminishes in more advanced cases [3,4]. Joint arthrodesis can alleviate pain but restricts joint mobility. PIP arthrotomy with Reg-Joint implantation offers pain relief while preserving mobility, though it may lead to joint instability and the persistent widening of joint contours [7]. Treatment involving autogenous grafts, with or without vascularization, shows promise but is restricted in its application. Vascularized autografts from toe joints demonstrate efficacy, particularly in pediatric cases [5]. Avascular autografts from the hamate bone are commonly utilized in fractures involving the distal part of the joint with dorsal displacement [6].

Arthroplasty is the gold standard after unsuccessful conservative treatment. Silicone prostheses have demonstrated superior outcomes in the literature. Surgeons favoring silicone implants note their ability to accommodate anatomical and surgical irregularities, albeit with poorer lateral stability. Silicone prostheses are widely applicable in PIP joint arthroplasty, utilizing various surgical approaches and implant types [3]. According to Yamamoto et al., volar approaches for silicone prostheses achieve optimal mobility outcomes, minimize postoperative complications, reduce the need for revisions, and mitigate postoperative joint flexion contractures. However, drawbacks include compromised lateral stability, reduced range of motion postoperatively, implant fractures, and potential material degradation [8].

The patients undergoing cementless hemiprosthesis implantation retain their PIP joint collateral ligaments, offering advantages over silicone prostheses. Enhanced lateral stability is particularly beneficial for younger patients. Additionally, this type of prosthesis facilitates greater postoperative mobility. However, drawbacks include a higher incidence of postoperative complications and a more limited range of motion in long-term follow-up [8,9].

Daecke et al. reported that grip strength in the patients with the CapFlex PIP hemiprosthesis was comparable to that of adjacent healthy fingers. Similar outcomes were observed in patients using silicone, titanium, or pyrocarbon anatomical prostheses. They also noted no significant difference in the long-term range of motion outcomes between PIP arthroplasty using silicone spacers and surface replacement prosthesis (SRP) made of titanium or pyrocarbon. All implants effectively manage long-term pain, which is the primary treatment goal [9]. Moreover, SRP prostheses, when placed with the preservation of the ligamentous apparatus, provide superior lateral stability, particularly beneficial for joints with a deformed ulnar condyle of the proximal phalanx and an ulnar deformation of the PIP joint [10]. According to Shirakawa and Shirota [11] and Daecke et al. [9], this type of prosthesis allows for a greater range of motion in the early postoperative period. However, SRP implants are associated with a higher incidence of postoperative complications, implant loosening, revision procedures, osteophyte formation in the long term, and a smaller range of motion in the long-term follow-up [8,9,11].

## Conclusions

The implantation of the CapFlex PIP prosthesis in a patient with post-traumatic degenerative disease restores the correct range of flexion movement, realigns the fourth radius of the hand, and effectively alleviates pain. However, this treatment method may entail a slight limitation in the range of extension motion in the joint.
